# Predictors of unsuccessful interim treatment outcomes of multidrug resistant tuberculosis patients

**DOI:** 10.1186/s12879-017-2746-5

**Published:** 2017-09-29

**Authors:** Muhammad Atif, Arslan Bashir, Nafees Ahmad, Razia Kaneez Fatima, Sehar Saba, Shane Scahill

**Affiliations:** 1 Department of Pharmacy, The Islamia University of Bahawalpur, Bahawalpur, Pakistan; 2 Faculty of Pharmacy and Health Sciences, University of Balochistan, Quetta, Pakistan; 3 Research Unit, National Tuberculosis Control Program of Pakistan, Islamabad, Pakistan; 4 Chest Disease Unit, Bahawal Victoria Hospital, Bahawalpur, Pakistan; 5 School of Management, Massey University, Auckland, New Zealand

## Abstract

**Background:**

Interim treatment outcomes at 6-months for multidrug-resistant tuberculosis (MDR-TB) treatment are among the most basic performance monitoring and key evaluation indicators in the Stop and End TB strategy of the World Health Organization (WHO). Therefore, this study was conducted to evaluate the interim treatment outcomes of MDR-TB patients in Pakistan.

**Methods:**

This study was conducted at the Programmatic Management Unit for Drug-resistance TB (PMDT) site of the National Tuberculosis Program (NTP), Pakistan. It is located in the Chest Disease Unit (CDU) of the Bahawal Victoria Hospital (BVH), Bahawalpur, Punjab, Pakistan. Data was collected between April 1, 2014 and December 31, 2015. The medical records, Electronic Nominal Recording Reporting System (ENRS) data and MRD-TB notification forms of the MDR-TB patients registered at the PMDT site were reviewed to obtain data. For reporting and calculation of interim treatment outcomes, standardized WHO methodology was adopted. Simple logistic regression analysis was used to examine the possible association between the dependent variable (i.e. unsuccessful interim treatment outcome) and selected socio-demographic and clinical variables.

**Results:**

A total of 100 drug-resistant TB (DR-TB) patients (all types) were registered during the study period. Out of these, 80 were MDR-TB patients for whom interim results were available. Out of the 80 MDR-TB cases, 48 (60%) were classified under the successful interim treatment outcome category. The remaining 40% had unsuccessful 6-month treatment outcomes and 12 (15%) patients died, while nine (11.3%) were lost to follow-up by six months. The final predictors of unsuccessful interim treatment outcomes were; being resistant to ofloxacin (AOR 3.23, 95% CI 0.96–10.89; *p*-value = 0.04), having above normal baseline serum creatinine levels (AOR 6.49, 95% CI 1.39–30.27; *p*-value = 0.02), and being culture positive at the second month of treatment (AOR 6.94, 95% CI 2–24.12; *p*-value = 0.01).

**Conclusions:**

Despite free treatment and programmatic efforts to ensure patient adherence, the high rate of unsuccessful interim treatment outcomes is concerning. The identified risk factors for unsuccessful interim treatment outcomes in the current study provides clinicians an opportunity to identify high-risk patients and ensure enhanced clinical management and greater treatment success rates.

**Electronic supplementary material:**

The online version of this article (10.1186/s12879-017-2746-5) contains supplementary material, which is available to authorized users.

## Background

A major obstacle in the successful control of tuberculosis (TB) is multidrug-resistant TB (MDR-TB), defined as mycobacterium strains resistant to both isoniazid and rifampicin. The management and treatment of MDR-TB is complex and it is difficult to achieve favorable treatment outcomes as compared to drug-sensitive TB, even under optimal circumstances. In-part, this is attributed to the lengthy treatment of MDR-TB patients with comparatively less effective, more toxic and costly regimens that contain combinations of first line (FLDs) and second line anti-TB drugs (SLDs) [1–3]. Moreover, scarcity of an evidence base from randomized controlled trials, inadequate number of SLDs, the absence of political commitment, the limited number of experts and laboratories and the sale of anti-TB drugs in the private sector of high-TB burden countries, have also contributed toward the unsuccessful treatment outcomes seen among MDR-TB patients [2–5].

MDR-TB treatment is usually in the duration of 18 to 20 months and at times longer. Therefore, final treatment outcomes can only be evaluated after two to three years of patient registration on the MDR-TB treatment register. During the course of treatment the National Tuberculosis Programme (NTP) managers and healthcare authorities/providers require an indication of the treatment effectiveness among the enrolled patients [6]. This is more important when a Programmatic Management Unit for Dug-resistance TB (PMDT) site is newly established. Therefore, the WHO has set various interim benchmarks for monitoring drug-resistant TB programmes in order to take appropriate and timely action to control the burden of TB [6]. Of these, sputum culture negativity during the early months of MDR-TB treatment is widely used as a reliable interim indicator of non-infectiousness (i.e. microbiological endpoint) and for tracking clinical progress and the effectiveness of treatment [7, 8]. Based on the assumed predictive value of sputum culture conversion for end TB treatment outcome, this is used as an early microbiological end point in phase II clinical trials of TB treatment [9]. Yew et al. have reported that sputum culture conversion within the first three months of treatment was predictive of complete cure in 100% of MDR-TB patients. Sputum culture conversion at two months of treatment has also been reported as a predictor of successful treatment outcomes among MDR-TB patients [10]. Similarly, those patients who did not achieve sputum culture conversion after two months of treatment were 2.7 times more likely to develop unsuccessful treatment outcomes than those who converted within the first two months of treatment [11]. A recently conducted study which included 1712 MDR-TB patients from two separate cohorts, also found statistically significant positive associations between sputum culture conversion at two months of treatment and successful outcomes among patients with unknown HIV status. However, the study reports that overall association of sputum culture conversion with a successful treatment outcome was significantly greater at six months [9]. In addition to sputum culture conversion, the proportion of patients who are deceased at six months is also commonly used as an interim indicator of early treatment response. Figures on loss to follow-up at six months due to a variety of reasons such as: symptoms relief, adverse drug reactions, patient’s lack of awareness about the total treatment duration [12] is also helpful in the assessment of adherence to treatment regimens, and overall performance of the programme [6] (refer to Additional file 1: Appendix S1 section for study schema).

Pakistan has the fourth highest MDR-TB burden of any country in the world and is the highest in the Eastern Mediterranean Region of the WHO (EMRO) [13]. The first National Drug Resistance Survey (DRS) of Pakistan was completed in September 2013. According to the results of that DRS, the MDR-TB prevalence amongst new patients of pulmonary TB (PTB) was 3.7% and 18.1% in retreatment PTB patients [14]. The comparatively high prevalence of MDR-TB in retreatment TB cases in Pakistan is suggestive of defective anti-TB treatment in the past, and could be attributed to divergent guidelines and practices, poor patient adherence to anti-TB treatment, over-the-counter (OTC) sales of medicines and the poor quality of anti-TB drugs in Pakistan [15]. This also generates concerns about the suitability of the WHO Category II regimen in Category I failures. According to the WHO guidelines, in a country with a high burden of drug-resistant TB such as Pakistan, it is recommended that patients who fail Category I treatment should be evaluated for drug resistance, rather than putting them on the Category II regimen [15, 16]. It is a grim reality that despite the current epidemiological picture of MDR-TB in Pakistan, the projected government health expenditures (GHE) for MDR−/TB remains at less than 1.5% of Gross Domestic Product (GDP) per capita. Further, this is the lowest amongst the high-TB burden countries [13]. To date, several studies from different areas of Pakistan have documented the number of incident cases and treatment outcomes of MDR-TB patients, with no evidence of uniformity in the results [3, 17–20]. Moreover, no study from Pakistan has reported interim treatment outcomes of MDR-TB patients. The authors are not aware of any study that has reported the factors associated with unsuccessful interim treatment outcomes. Besides this, previous studies have not evaluated treatment outcomes in a cohort of patients registered at the newly established PMDT site of the Bahawal Victoria Hospital; that serves a large population of the Southern part of the Punjab province of Pakistan. Considering the recommendations of the WHO and the gaps in the existing literature, it is important to conduct cohort analyses of all MDR-TB patients in the local setting. Therefore, this study was conducted to evaluate the interim treatment outcomes of MDR-TB patients’ registered at a PMDT site of the NTP (Pakistan) established at Bahawalpur, Punjab, Pakistan. A vigilant analysis of the management and treatment status of MDR-TB patients, with an emphasis on the predictors of unsuccessful interim treatment outcomes, provides some understanding of progress and the way forward towards achieving the goal of a treatment success rate ≥ 90% for TB.

## Methods

### Study setting

This study was conducted at one of the PMDT sites of the NTP (Pakistan) established in the Chest Disease Unit (CDU) of the Bahawal Victoria Hospital (BVH), Bahawalpur, Punjab, Pakistan. At the time of the study, BVH was a 1600 bed, fully equipped, tertiary-care hospital with all medical and surgical specialties available, serving a large number of patients in the Southern Punjab region [21, 22]. Approximately 450 medical doctors and more than 25 pharmacists work in 10 departments, which consist of 27 units of various specialties and sub-specialties. The CDU of BVH is a long established unit that is well-equipped and provides healthcare services to both in and out-patients suffering from a range of chest-related diseases. At the CDU, 8 to 10 general physicians, 5 to 6 chest specialists and two pharmacists provide routine care to patients. Between 35 and 40 TB patients (all forms) visit the CDU daily to take their TB therapy. In March 2014, with the support of the NTP through the Global Fund, a separate section for the management of MDR-TB patients was established in the CDU as a PMDT site [21]. At that time approximately 161 active MDR-TB cases were managed in that section of the CDU. This MDR-TB section has its own designated team of physicians, pharmacist, psychiatrist, treatment coordinator, laboratory technician and other clerical staff members. This section also has a well-established affiliated laboratory for sputum smear microscopy and rapid drug susceptibility testing (DST). The radiology and pathology departments of BVH also provide routine investigation services to MDR-TB patients.

### Study population

**Fig. 1:**
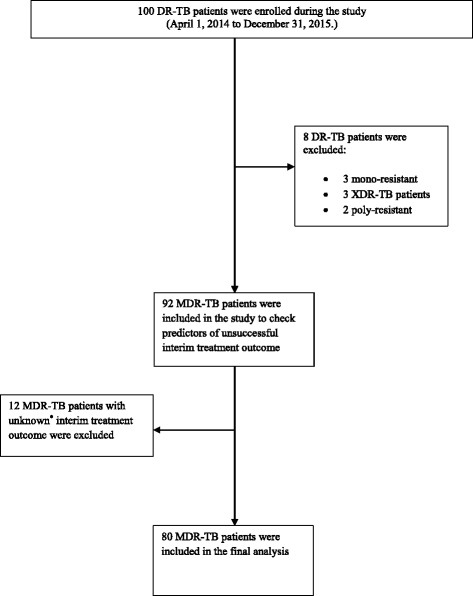
Enrolment, inclusion and exclusion of the study patients

This was a descriptive, retrospective cohort study of all MDR-TB patients diagnosed and registered at the study site between April 1, 2014 and December 31, 2015. Patients were considered eligible for inclusion in the study if they had a M. *tuberculosis* isolate with resistance to at least isoniazid and rifampin and had been started on a MDR-TB treatment regimen with second-line anti TB drugs using the programmatic management of drug-resistant TB (PMDT) strategy during the study period irrespective of age, gender, race, ethnicity and comorbidity. Patients with a previous history of MDR-TB treatment or extra pulmonary MDR-TB, mono-, poly and XDR-TB patients and those for whom interim indicator results were not available were excluded. Additionally patients on MDR- and XDR-TB treatment regimens and later found not to have MDR-TB and XDR-TB were also excluded. During the study period, a total of 100 DR-TB patients (all types) were registered at the study site. Out of these, 80 were MDR-TB patients for whom interim results were available. Therefore, this subset of patients was included in the study for the final analysis. Figure 1 outlines the stepwise enrolment, inclusion and exclusion of the study patients.

### Bacteriology and drug susceptibility testing

According to the NTP guidelines, “presumptive TB cases” were subjected to an in-depth clinical interview at the TB out-patient department to identify DR-TB risk factors. Patients with possible DR-TB were then directed to the PMDT site for initial evaluation. At the site, two sputum samples (spot and morning from the next day) were obtained from the patients. The spot specimen was examined through direct smear microscopy for the presence of acid fast bacilli (AFB) using the Ziehl-Neelsen stain. The rapid drug susceptibility testing (DST) (i.e. Xpert MTB/RIF®) assay was also performed to detect rifampicin resistance (RR).

After positive smear microscopy and RR detected through rapid drug susceptibility Xpert MTB/RIF assay, the morning or spot specimen (if no morning specimen was obtained) was sent to the National Reference Laboratory (NRL) in Islamabad via a courier service in the cold chain system, within 72 h. In the NRL direct sputum smear microscopy for AFB strains using Ziehl-Neelsen stain and culture examination using the modified Kudoh method were performed. Moreover, drug susceptibility testing (DST) using the agar proportion method on enriched Middle-brook 7H10 medium against FLD (i.e. rifampicin, isoniazid, streptomycin and ethambutol) and SLD (i.e. ofloxacin, amikacin, kanamycin, ethionamide and capreomycin) was also performed. DST for pyrazinamide was performed using BACTEC™ MGIT™ (BD) in accordance with the manufacturer’s instructions. DST was performed at baseline and repeated whenever deemed necessary, while AFB sputum smear and culture were performed every month.

### Treatment protocol

Patients found to be smear positive and resistant to rifampicin were then registered to receive the appropriate treatment protocol in compliance with the national guidelines [23]. An empirical regimen consisting of amikacin/kanamycin/capreomycin + levofloxacin + ethionamide + cycloserine + pyrazinamide + vitamin B6 was provided to all patients, except those who had a history of SLD use. In patients with a documented history of SLD use, treatment was initiated by adding para-amino salicylic acid (PAS) to the above stated regimen. After the availability of DST results, the patients received a tailored and individualized treatment regimen using at least four drugs for which the patient had confirmed susceptibility. Maximum recommended doses of drugs were prescribed based upon patient body weight. Pyrazinamide, ethambutol and fluoroquinolones were administered once daily to increase their efficacy, while ethionamide/prothionamide, p-aminosalicylic acid and cycloserine were provided in split doses to reduce adverse drug reactions. Patients were treated for at least 20 months with 18 months after culture conversion, defined as two consecutive negative sputum cultures taken at least 30 days apart, following initial positive culture. Injectable SLDs were administered to patients for a minimum of eight months, with at least four months of negative culture (without any positive, contaminated or missing culture results in between). All patients were treated on an ambulatory basis and were evaluated monthly. Trained support for monitoring of adherence was provided. For each observed dose, the treatment supporter marked the patient’s treatment card. The clinician also assessed patient adherence during monthly visits by inspecting the treatment card. Treatment adherence was also ensured by a home DOTS linkage (HDL) facilitator who made home visits, linking the patients, the PMDT unit, the District TB Officer (DTO) and the nearest health care center.

Adequate and immediate management of any reported adverse drug reactions was undertaken to minimize the risks of treatment interruption, reduced adherence and associated morbidity and mortality. Monthly social support in the form of food baskets and a transport allowance was also provided to patients and their treatment supporters. Patients who were lost to follow-up for greater than four weeks and did not come back to receive medicines and have routine investigations were contacted first via telephone, then through tracing by a treatment-coordinator.

### Data collection

The medical records, Electronic Nominal Recording Reporting System (ENRS) data and MDR-TB notification forms of the participants were reviewed to obtain socio-demographic, clinical, microbiological and treatment-related data. The socio-demographic data included; age, sex, marital status, area of residence, household size, smoking and employment status. The clinical data included baseline body weight, hemoglobin level, white blood cell level, bilirubin level, creatinine level, number and type of co-morbidities, history of streptomycin use, history of SLD use and family history of TB.

### Calculation and reporting of interim treatment outcomes

Interim treatment outcomes were analyzed according to criteria defined in the “Companion Handbook to WHO Guidelines for the Programmatic Management of Drug-Resistant Tuberculosis” [6] (refer to Additional file 2: Appendix S2 section for the detail of interim indicators used for monitoring RD-TB programmes). To check the predictors of unsuccessful interim treatment outcome, interim indicators for monitoring of the DR-TB programmes were classified into two groups. Culture results negative at six months were grouped under a successful interim treatment outcome, whereas death, loss to follow-up, and positive culture at six months were all categorized as unsuccessful interim treatment outcomes. The patients on MDR- and XDR-TB treatment regimens that were found not to have MDR-TB and XDR-TB, respectively, were not categorized and were excluded from the study.

### Data management and analysis

All statistical analysis was undertaken using the SPSS (Statistical Package for the Social Sciences, version 20, Armonk, NY: IBM Corp.) for Windows™. All continuous variables were reported as means and standard deviations (SD) or as median and range after assessing the normality of the data by applying the Kolmogorov-Smirnov test [24]. Categorical variables were described using counts and proportions (%). Simple logistic regression analysis was used to examine the possible association between the dependent variable (i.e. unsuccessful interim treatment outcome) and selected socio-demographic and clinical variables. All factors considered in the univariate analysis were based on literature review and suggestions from the clinical team. The crude odds ratio (OR), 95% confidence interval (CI) for beta, standard error and *p*-value were reported for each predictor in the univariate analysis. Only statistically significant variables in the univariate analysis were entered into multiple logistic regression analysis in-order to predict the final independent factors. Likewise, the adjusted odds ratio (AOR), 95% CI, beta, standard error and *p*-value were reported for each predictor. The model fit was assessed by chi-square, degrees of freedom and *p*-value. Pseudo R square values were included to provide information about the percentage of variance explained by the model. The significance of the statistical tests was taken at a p-value of < 0.05 [24].

## Results

### Baseline characteristics of the patients

Of the 80 MDR-TB patients, the mean age was 37.2 (SD = 17.48) years and the majority (*n* = 52, 65%) were within working age (18–54 years). Residents from rural areas constituted the largest proportion (*n* = 69, 86.3%) of patients. Clinically, two-thirds had above-normal baseline levels of hemoglobin, bilirubin and creatinine. Twenty-two (27.5%) patients had one or more comorbidities and surprisingly, all patients were HIV negative. A total of 28 (35%) patients had a history of streptomycin use and seven (8.8%) of SLDs. Table 1 provides a full description of the baseline characteristics of the participants.

**Table 1:** Characteristics of the Patients (*n* = 80)

Characteristics	Patients *n* (%)
Sex	
Male	43 (53.8)
Female	37 (46.3)
Age group (years)	
0–4	0 (0)
5–14	5 (6.3)
15–24	19 (23.8)
25–34	17 (21.3)
35–44	11 (13.8)
45–54	8 (10)
55–64	13 (16.3)
≥ 65	7 (8.8)
Marital status	
Unmarried	26 (32.5)
Married	51 (63.8)
Widow/divorced	3 (3.8)
Residence	
Urban	11 (13.8)
Rural	69 (86.3)
Household size	
≤ 7	38 (47.5)
> 7	34 (42.5)
Unknown	8 (10)
Smoking status	
Non-smoker	58 (72.5)
Ex-smoker	18 (22.5)
Active smoker	4 (5)
Employment status	
Employed	35 (43.8)
Unemployed	45 (56.3)
Baseline weight (in kg)	
< 40	37 (46.3)
≥ 40	43 (53.8)
Hb level^*^	
Normal	23 (28.8)
Below normal	57 (71.3)
WBCs level^*^	
Normal	44 (55)
Above normal	36 (45)
Bilirubin level^*^	
Normal	65 (81.3)
Above normal	15 (18.8)
Creatinine level^*^	
Normal	61 (76.3)
Above normal	17 (21.3)
Unknown	2 (2.5)
Comorbidities	
No	58 (72.5)
Yes	22 (27.5)
Type of comorbidities	
Diabetes Mellitus	13 (16.3)^†^
Hepatitis	10 (12.5)^†^
Others^‡^	3 (3.75)^†^
History of streptomycin use	
No	52 (65)
Yes	28 (35)
History of SLD use	
No	73 (91.3)
Yes	7 (8.8)
Family history of TB	
Absent	60 (75)
Present	20 (25)

^*^Normal ranges: Hb = Male >13 g/dl, Female >11.5 g/dl; WBCs = > 11,000/mm3; Creatinine = Male <1.1 mg/dl, Female < .9 mg/dl; Bilirubin = ≤ 1 mg/dl

^†^Patients with one or more comorbidities

^‡^Includes patient with ischemic heart disease, chronic obstructive pulmonary disease and systemic lupus erythematosus

Hb = Hemoglobin; SLD = Second Line Drugs; TB = Tuberculosis; WBCs = White Blood Cells

### Drug resistance patterns

A high level of drug resistance was observed among the study participants. The patients were resistant to a median of four drugs (range 2–7), while over one-quarter (*n* = 21, 26.3%) of the patients were resistant to more than four drugs. Thirty-three (40%) patients were resistant to more than three FLDs, while 12 (12.5%) were resistant to all five FLDs. Notably; exactly half of the participants were resistant to streptomycin. Thirty-one (38.8%) patients were resistant to at least one SLD, while only two (2.5%) were resistant to two SLDs. The most common SLD, to which patients were resistant was ofloxacin (31 out of 33 SLD resistant patients). Thirty-three (41.3%) patients were resistant to both FLD and SLD simultaneously, whereas 22 (27.5%) were resistant to FLD alone (Table 2).

**Table 2:** Drug resistance pattern of studied patients (n = 80)

Variable	Patients *n* (%)
Resistance to FLD	
*Names*	
Resistance to HR	7 (8.8)
Resistance to HRE	6 (7.5)
Resistance to HRES	19 (23.8)
Resistance to HRESZ	10 (12.5)
Resistance to HRS	10 (12.5)
Resistance to HRZ	1 (1.3)
Resistance to HRZS	1 (1.3)
Resistance to HREZ	3 (3.8)
Results unavailable^*^	23 (28.8)
*Numbers*	
2	7 (8.8)
3	17 (21.3)
4	23 (28.8)
5	10 (12.5)
Results unavailable^*^	23 (28.8)
Resistance to SLD	33 (41.3)
*Names*	
Resistance to Ofx	31 (38.7)
Resistance to Eto	1 (1.3)
Resistance to Km	1 (1.3)
Resistance to Ofx + Z	1 (1.3) ^†^
Resistance to Ofx + Eto	1 (1.3) ^†^
Results unavailable^*^	47 (58.7)
*Numbers*	
1	31 (38.7)
2	2 (2.6)
Results unavailable	47 (58.7)

^*^ Results were not documented due to one of the following reasons: drug susceptibility testing results awaited, culture contamination, no growth of culture, leakage of sample during transportation and no sputum production by the patients

^†^ of the 31 Ofx-resistant patients

E = Ethambutol; Eto = Ethionamide; FLD = First Line Drugs; H = Isoniazid; Km = Kanamycin; Ofx = Ofloxacin; R = Rifampicin; S = Streptomycin; SLD = Second Line Drugs; Z = Pyrazinamide

### Treatment regimen during the intensive phase

At the start of the study before the availability of DST results, a standardized treatment regimen according to the NTP guidelines was provided to all registered MDR-TB patients whose diagnosis was done by Xpert MTB/RIF. After availability of DST results, the treatment regimen was modified for 52 (65%) patients. The most common modification was addition of p-aminosalicylic acid (in 27.5% patients) as a clean drug, due to presence of resistance to ofloxacin, followed by the addition of ethambutol (in 17.5% patients) and pyrazinamide (in 5% patients). During the treatment, amikacin was replaced with capreomycin, due to the associated ototoxicity among three (3.8%) patients.

During the intensive phase, a median of seven drugs (range 5–11) were administered to the participants and all received levofloxacin, ethionamide and cycloserine. Additionally, pyrazinamide was given to 79 (98.8%) patients and amikacin to 70 (87.5%) patients.

### Adverse events during the intensive phase

A total of 19 patients (23.75%) experienced one or more adverse events. The most prevalent adverse event was gastrointestinal disturbance followed by psychiatric disorders and hearing disturbances. The occurrence of adverse event with life threatening potential was rare. Hepatotoxicity and nephrotoxicity respectively developed in two and three patients, respectively (Table 3).

**Table 3:** Adverse events associated with the multidrug-resistant tuberculosis therapy during first six months of the treatment (n = 80)

Adverse events	Patients^*^ *n* (%^†^)
Gastrointestinal effects	9 (11.3)
Arthralgia	3 (3.9)
Psychiatric disorders	7 (8.8)
Hearing disturbances	6 (7.5)
Renal toxicity	3 (3.8)
Hepatotoxicity	2 (2.5)
Dermatologic reactions	3 (3.9)
Peripheral neuropathy	2 (2.5)
Hematological reactions	1 (1.3)
Others	6 (7.5)

^*^ Patients with one or more adverse drug reactions

^†^ 80 Multidrug-resistant tuberculosis patients

### Successful and unsuccessful interim treatment outcomes

Out of the 80 MDR-TB cases receiving the treatment regimen, over half (*n* = 48, 60%) were classified under the successful interim treatment outcome category. In terms of unsuccessful interim treatment outcomes, 12 (15%) patients died, while nine (11.3%) were lost to follow-up at the six month point. Table 4 illustrates the proportion of patients in the successful and unsuccessful interim treatment outcome categories.

**Table 4:** Interim treatment outcomes of the patients^*^ (*n* = 80)

Treatment outcomes	Patients *n* (%)	Total *n* (%)
Successful		
Negative culture by six months	48 (60)	48 (60)
Unsuccessful		
Died by six months	12 (15)	32 (40)
Lost to follow-up by six months	9 (11.3)	
Positive culture by six months	11 (13.8)	

^*^Multidrug-resistant patients included in the final analysis to check predictors of unsuccessful interim treatment outcomes

At the time of analysis, among the 48 patients with interim successful treatment outcomes, 37 were still under treatment with persistent culture negative status, whereas final treatment outcomes were available for 11 patients. Among these 11 patients, 6 were declared cured, three died, one was transferred out and one lost to follow up. Among the 32 patients with unsuccessful interim treatment outcomes, 6 patients were still under treatment, whereas final treatment outcomes were available for 26 patients. Among these 26 patients, 15 had died, eight were lost to follow up and three were transferred out (Table 5).

**Table 5:** Interim and final treatment outcomes cross-tabulation

	Final outcomes at the time of analysis (*n, %*)					Total
	Still under treatment	Died	Cured	Transfer out	Lost to follow-up	
Successful interim treatment outcomes n (%)	37 (77.1)	3 (6.3)	6 (12.5)	1 (2.1)	1 (2.1)	48
Unsuccessful interim treatment outcomes n (%)	6 (18.8)	15 (46.9)	0 (0)	3 (9.4)	8 (25)	32
Total n (%)	43 (53.8)	18 (22.5)	6 (7.5)	4 (5)	9 (11.3)	80

### Predictors of unsuccessful interim treatment outcomes

In the univariate analysis, unsuccessful interim outcomes demonstrated statistically significant association with patient’s age (25–34 years) (*p*-value = 0.024, OR = 3.667), above normal serum creatinine at baseline visit (*p*-value = 0.015, OR = 4.053), history of streptomycin use (*p*-value = 0.007, OR = 3.812), ofloxacin resistance (*p*-value = 0.010, OR = 3.462) and sputum culture positivity at two months of treatment (*p*-value = 0.005, OR = 9.545) (Table 6).

**Table 6:** Predictors of unsuccessful interim treatment outcomes: simple logistic regression analysis

Independent Variable*	Interim Treatment outcomes		B	S.E	*p*-value	(OR) 95% CI
	Unsuccessful (*n* = 32)	Successful (*n* = 48)				
Age 25–34 (years)						
No	21	42				1
Yes	11	6	1.299	0.574	**.024**	3.667 (1.191, 11.285)
Weight^†‡^	–	–	−0.027	0.020	.187	0.974 (0.936, 1.013)
Hb level^‡^						
Normal	6	17				1
Below normal	26	31	0.866	0.544	.112	2.376 (0.818, 6.905)
Creatinine level^‡^						
Normal	19	42				1
Above normal	11	6	1.399	0.578	**.015**	4.053 (1.306, 12.580)
Previous TB episodes^†^	–	–	0.456	0.315	.148	1.577 (0.850, 2.927)
Months of sickness with TB						
< 12	21	24				1
≥ 12	11	24	−0.647	0.471	.170	0.524 (0.208, 1.319)
Previous TB treatment outcome failed						
No	30	40				1
Yes	2	8	−1.099	0.827	.184	0.333 (0.066, 1.685)
History of streptomycin use						
No	15	37				1
Yes	17	11	1.338	0.493	**.007**	3.812 (1.449, 10.026)
Number of first line drugs with resistance^¶^						
< 4	11	8				1
≥ 4	14	22	−0.770	0.577	.182	0.463 (0.149, 1.434)
Ofloxacin resistance						
Present	14	35				1
Absent	18	13	1.242	0.482	**.010**	3.462 (1.345, 8.906)
Sputum grading^‡¶^						
Low	17	35				1
High	10	9	0.827	0.546	.130	2.288 (0.784, 6.675)
Positive culture after two months of treatment						
No	11	40				1
Yes	21	8	2.256	0.537	**< 0.01**	9.545 (3.331, 27.354)

*p*-value less than 0.05 in bold

* Variables tested but not included in this Table because the *p*-value was greater than .20: sex, residence, being married, smoking status, concurrent comorbidities, family history of TB, number of resistant drugs, resistant to any second line anti-TB drug, base line sputum results, baseline levels of hemoglobin, serum glutamate pyruvate transaminase, sputum positive after two months of treatment, adverse events observed during six months of treatment

^†^Continuous variable,

^‡^Baseline values

^¶^Variables with greater than 10% missing values

AFB = Acid Fast Bacilli; Hb = Hemoglobin; HPF; High Power Field; MDR-TB = Multidrug-Resistant TB; TB =Tuberculosis

After adjusting for the determinants of unsuccessful interim treatment outcomes in the multivariate analysis, the factors which remained statistically significantly associated with unsuccessful interim treatment outcomes were; ofloxacin resistance, above normal baseline serum creatinine level, and being culture positive at the second month of treatment (Table 7).

**Table 7:** Predictors of unsuccessful interim treatment outcome: multiple logistic regression analysis

Independent variable	B	S.E	*Sig.*	AOR	95% CI for Exp (B)
Resistance to ofloxacin	1.173	0.620	**.044**	3.232	1.959, 10.891
Above normal serum creatinine level (baseline)	1.87	0.786	**.017**	6.490	1.392, 30.266
Culture positivity at second month of the treatment	1.938	0.635	**.002**	6.944	1.999, 24.12
History of streptomycin use	0.948	0.667	.155	2.581	0.699, 9.531
Age group 25–34 (years)	1.232	0.761	.105	3.429	0.772, 15.228

*p*-value less than 0.05 in bold. Model summary = Chi square (34.574), df (5), *p* < 0.0005; Nagelkerke R Square (0.486); Hosmer and Lemeshow Chi square test (7.305), *p* = 0.294

## Discussion

This study set out to evaluate the interim treatment outcomes of MDR-TB patients in in South Punjab province of Pakistan. By the end of the study period, interim treatment outcomes were available for 80 MDR-TB cases, of whom 32 (40%) were classified under the unsuccessful interim treatment outcome category. In terms of unsuccessful interim treatment outcome, 12 (15%) patients died, while nine (11.3%) were lost to follow-up by six months. Based upon these results, it is evident that the study site has not achieved the treatment success rate set out in the goals of the Stop TB Strategy (75%), End TB strategy (90%) and the United Nations Sustainable Development Goals (80%) [13].

This failure is contributed by over one-quarter of the patients who died or were lost to follow-up. In accordance with the findings of previous reports [2, 25–27] there was a high loss to follow-up rate (11.3%) in the current study. This is alarming because treatment was free and there was a consistent and reliable supply of drugs, monthly food and transport allowances for both patients and treatment supporters, health education and counselling by the pharmacist and psychiatrist, home visits by the treatment coordinator and weekly checkups by the medical officer at the nearest health center. This support was provided to all patients, and still the “lost to follow up” rate is sub-optimal. Additionally, in the case that a patient defaulted for more than four weeks, patient tracing was also performed. Despite these efforts the literature points to the following reasons for loss of patients to follow-up; toxicity of the MDR-TB treatment regimen, poor disease knowledge, rural domicile, lower socioeconomic status, lower education levels, previous history of anti-tuberculosis treatment, lack of family support and dissatisfaction with health care worker attitudes [12, 26, 28, 29]. Strengthening of the Pakistan healthcare system with proper training of treatment supporters and comprehensive counseling of patients with “patient friendly” follow up services may help to reduce the rate of loss to follow-up in Bahawalpur.

The mortality rate (15%) observed in this study is comparable to previous studies from Pakistan, Uzbekistan and the Russian Federation, [3, 27, 30]. In contrast, lower mortality rates have been reported elsewhere [2, 25, 26, 31]. In Bulgaria, during 2009–2010, mortality rates of 38% and 53.2% were observed among patients who were lost to follow-up [32]; therefore, masking of deaths due to high rates of loss to follow up of patients could be a reason for the lower mortality rates in the aforementioned studies. The higher mortality rate in this study, based upon the literature, could be attributed to delayed detection of the disease or treatment initiation, lower education, greater number of previous TB episodes, history of diabetes, poor bacteriologic response and inadequate treatment [32]. Therefore, new strategies for appropriate MDR-TB detection and management should be implemented at the study site.

An alarming finding of our study was positive association between ofloxacin resistance and the unsuccessful interim treatment outcome. This finding is of major concern and highlights the importance of fluoroquinolones in MDR-TB treatment regimens and this study aligns with previous reports from Pakistan [3, 12, 17, 18] and other countries [26, 33] supporting an increase in fluoroquinolone resistant M. *tuberculosis* strains. This high-level of resistance to ofloxacin in the Pakistani population is clearly concerning, and may be directly attributed to over the counter (OTC) sale of antibiotics and extensive, unregulated and irrational use of fluoroquinolones in particular; especially in pneumonia and uncomplicated respiratory-tract infections [34].

A very high rate of ofloxacin resistance was observed in the cohort of patients in this study. The previously reported high cross-resistance between ofloxacin and levofloxacin (87%) among MDR-TB patients in five MDR-TB high burden countries including Pakistan, calls into question the national guidelines’ recommendation of indiscriminate use of levofloxacin in treating MDR-TB patients [23]. In order to improve MDR-TB patients’ adherence and treatment outcomes, the WHO has recently recommended a new, shorter regimen for the treatment of MDR-TB patients. This regimen comprised of 4–6 months of kanamycin, moxifloxacin, prothionamide, clofazimine, pyrazinamide, ethambutol and high-dose isoniazid followed by 5 months of moxifloxacin, clofazimine, pyrazinamide and ethambutol [35] has produced excellent results under research conditions, in various settings [36, 37]. As the new regimen contains moxifloxacin instead of levofloxacin, the low prevalence of moxifloxacin resistance among rifampicin resistant TB patients in Pakistan (13.8% at 0.5 μg/ml which reduced further to 1.4% at 2 μg/ml), and very low rate of cross resistance between moxifloxacin and ofloxacin (7%) [33] makes the new regimen the most suitable option for treating ofloxacin resistant MDR-TB patients, and should be adopted without further delay [38]. Furthermore, the rational use of fluoroquinolones must be enacted immediately, to assist in preserving its effectiveness and the ongoing use in MDR-TB treatment regimens.

Sputum culture negativity during the early months of MDR-TB treatment (especially after two months) - is widely adopted as a reliable interim indicator of non-infectiousness (i.e. microbiological endpoint) and for monitoring and predicting ongoing progress, effectiveness and ultimately success of treatment [7, 8]. Successful treatment outcomes have been reported in MDR-TB patients who achieved sputum culture negativity after two months of treatment [10, 39]. In our study, sputum culture positivity even after two months of MDR-TB treatment was positively associated with unsuccessful interim treatment outcomes. Similarly unsuccessful treatment outcomes have been reported in MDR-TB patients with positive sputum culture results after two months of treatment [40]. According to a study from Hong Kong, culture negativity after two months of MDR-TB treatment was 100% and 52.3% prognostic of treatment success and failure, respectively [10]. In addition, a secondary analysis of data from two observational cohort studies and a study from the Dominican Republic has reported better treatment outcomes in MDR-TB patients with negative sputum culture results after two months of treatment [11, 41]. The results from our study also advocate that early negativity of sputum culture is a positive predictor of successful interim treatment outcomes, and vice versa. This may help clinicians in the early detection of patients who are at greater risk of unsuccessful treatment outcomes. Moreover, our study results also advance credibility for the clinical utility of checking sputum cultures as a method of evaluating the treatment effectiveness.

In the current study, at baseline visit, a notable proportion of patients had above normal serum creatinine levels. Upon cross tabulation, the authors found a statistically significant positive association between previous use of aminoglycosides and the above normal serum creatinine level at baseline visit (*p*-value = 0.038). This finding supports the previously reported nephrotoxic effects of these drugs [42, 43]. An important and unique finding of our study was the positive relationship between the baseline above normal serum creatinine level and unsuccessful interim treatment outcomes. Injectable SLDs play a central role in the management of MDR-TB [44, 45]. Resistance to injectable SLDs has been widely reported as a predictor of unsuccessful treatment outcomes among MDR-TB patients [45, 46]. Nephrotoxicity in MDR-TB patients is usually managed by reducing the dose or frequency of injectable SLD administration or suspending the causative agent [6, 47, 48]. The authors suspect that for preventing further damage to the kidney in patients with nephrotoxicity at the baseline visit, initiating treatment with low doses of injectable SLDs in this group of patients might result in the suboptimal concentrations and in turn, interim unsuccessful treatment outcomes. These patients require more detailed tailoring and significant alterations in their treatment regimens and this may complicate therapy and reduce adherence to their MDR-TB treatment. Therapeutic drug monitoring in this group of patients would provide an opportunity for clinicians to individually manage each patient’s anti-TB therapy.

As with any study, this research has raised questions in the process of answering others. From a clinical viewpoint studies are needed to confirm the finding of positive association between above normal baseline serum creatinine levels and unsuccessful interim treatment outcomes. From a health system viewpoint, there are deficiencies in Pakistan with a lack of clinical hospital pharmacists [49] and there is a need to develop dose modification protocols and incorporate the role of the hospital pharmacist into the adjustment of anti-TB drug dosing; especially in patients with previously compromised renal function. This could be evaluated as an intervention study. Likewise, the high level of loss to follow-up needs to be further explored, based on the fact that comprehensive support was provided to the participants in this study and it is important to understand in a qualitative way why this follow-up system has failed so dramatically [12].

Regardless of the fact that this study was performed with a robust and standardized methodology adopted from the WHO, our study still has a number of limitations. First, findings of this study may not be generalized for the whole of Pakistan because the sample was drawn from a single PMDT site in the southern part of the Punjab province of Pakistan. Nevertheless, the site is well established with all healthcare facilities available, and policies and practices at this site are expected to be comparable to other PMDT sites in Punjab specifically, but also Pakistan in general. Second, due to the retrospective nature of the study, (some important data was missing in the patient notification forms) it was not possible to include findings of baseline lung cavitation which has been previously reported as an important predictor of unsuccessful treatment outcome in TB [3, 32].

## Conclusion and recommendations

Despite free treatment and programmatic efforts to improve patients’ adherence with MDR-TB treatments, the high rate of unsuccessful interim treatment outcomes in the current cohort of Pakistani patients is concerning. Educational and psychosocial support interventions and decentralizing of treatment services may help to decrease the loss to follow-up rate at the study site. Having a focus on enhanced clinical management to patients with ofloxacin resistance and above normal serum creatinine levels at the baseline visit may improve the treatment outcomes at the center. In future, wide scale studies are needed to confirm the finding of a positive association between above normal baseline serum creatinine levels and unsuccessful interim treatment outcomes in MDR-TB patients.

## Additional files

Additional file 1: Appendix S1. Study schema. (DOCX 45 kb)

Additional file 2: Appendix S2. Interim Indicators for Monitoring Drug-Resistant Tuberculosis Programmes. (DOCX 27 kb)
