# Supplementary material for: Predictors of unsuccessful interim treatment outcomes of multidrug resistant tuberculosis patients
Source: BMC Infect Dis. 2017 Sep 29;17:655. doi: 10.1186/s12879-017-2746-5 (PMC5622487; doi:10.1186/s12879-017-2746-5)
Supplement: Supplementary file 1 — Appendix S1. Study schema. (DOCX 45 kb) [file 12879_2017_2746_MOESM1_ESM.docx]

**Additional file 1: Appendix S1 (Study Schema)**

Figure: *Study schema*

Performance of National Tuberculosis Program, Pakistan

An early indication of the final treatment outcomes

Treatment effectiveness

Treatment response

Treatment progress

Interim treatment outcomes

Successful interim treatment outcome:

- Culture negativity after six month of the treatment

Treatment regimen (intensive phase)

Adverse drug reactions (during first six months of the treatment

Drug-resistance pattern (DST)

Sputum smear and culture outcomes (baseline and after two months of the treatment)

Unsuccessful interim treatment outcome:

- Culture positivity after six month of the treatment
- Patients died after six months of the treatment
- Patients lost to follow-up after six months of the treatment

Baseline clinical characteristics of the patients

Socio-demographic characteristics of the patients
