# Supplementary material for: Predictors of unsuccessful interim treatment outcomes of multidrug resistant tuberculosis patients
Source: BMC Infect Dis. 2017 Sep 29;17:655. doi: 10.1186/s12879-017-2746-5 (PMC5622487; doi:10.1186/s12879-017-2746-5)
Supplement: Supplementary file 2 — Appendix S2. Interim Indicators for Monitoring Drug-Resistant Tuberculosis Programmes. (DOCX 27 kb) [file 12879_2017_2746_MOESM2_ESM.docx]

| **Additional file 2: Appendix S2**  *Interim Indicators for Monitoring Drug-Resistant Tuberculosis Programmes* | |
| --- | --- |
| **Indicator number and name** | **Indicator calculation** |
| **1:**Number (%) of confirmed RR-/MDR-TB cases on MDR-TB treatment regimen with negative culture by six months | Numerator: Number of confirmed pulmonary RR-/ MDR-TB cases registered and started on a prescribed MDR-TB treatment with negative results for culture in month 6 of their treatment. Denominator: Number of confirmed RR-/ MDR-TB cases registered and started on treatment for MDR-TB during the period of assessment |
| **2:**Number (%) of confirmed RR-/MDR-TB cases on MDR-TB treatment regimen that died by six months | Numerator: Number of confirmed pulmonary RR-/ MDR-TB cases registered and started on a prescribed MDR-TB treatment who died of any cause by the end of month 6 of their treatment.  Denominator: Number of confirmed RR-/ MDR-TB cases registered and started on treatment for MDR-TB during the period of assessment |
| **3:**Number (%) of confirmed RR-/MDR-TB cases on MDR-TB treatment regimen that lost to follow-up by six months | Numerator: Number of confirmed pulmonary RR-/ MDR-TB cases registered and started on a prescribed MDR-TB treatment who were lost to follow-up by the end of month 6 of their treatment. Denominator: Number of confirmed RR-/ MDR-TB cases registered and started on treatment for MDR-TB during the period of assessment |
| **4:**Number of patients started on MDR-TB treatment regimen found not to have MDR-TB | Number of confirmed pulmonary RR-/ MDR-TB cases registered and started on a prescribed MDR-TB treatment and later found not to be RR-/ MDR-TB |
| **5:**Number of patients started on XDR-TB treatment regimen found not to have XDR-TB | Number of confirmed pulmonary RR-/ MDR-TB cases registered and started on a prescribed MDR-TB treatment and later found not to be XDR-TB. |
| Note: First three indicators also included XDR-TB cases that started the prescribed treatment with second line drugs  MDR-TB = Multidrug-Resistant TB; RR-RB = Rifampicin Resistant TB; XDR-TB = Extensively Drug-Resistant TB.  Adapted from: Companion Handbook to WHO Guidelines for the Programmatic Management of Drug-Resistant Tuberculosis, 2014. | |
